# Acute *Garcinia mangostana* (mangosteen) supplementation does not alleviate physical fatigue during exercise: a randomized, double-blind, placebo-controlled, crossover trial

**DOI:** 10.1186/s12970-016-0132-0

**Published:** 2016-05-04

**Authors:** Chih-Wei Chang, Tzu-Zung Huang, Wen-Hsin Chang, Yi-Chun Tseng, Yu-Tse Wu, Mei-Chich Hsu

**Affiliations:** School of Pharmacy, Kaohsiung Medical University, 100, Shih-Chuan 1st Road, Kaohsiung, 80708 Taiwan; Department of Sports Medicine, Kaohsiung Medical University, 100, Shih-Chuan 1st Road, Kaohsiung, 80708 Taiwan

**Keywords:** α-mangostin, Hydroxycitric acid, Exercise, Exhaustion, Muscle dynamic stiffness, Blood biochemical

## Abstract

**Background:**

The purple mangosteen (*Garcinia mangostana*), known as the "queen of fruit," is widely consumed and unique not only because of its outstanding appearance and flavor but also its remarkable and diverse pharmacological effects. The aim of the present study is to evaluate the effect of acute mangosteen supplementation on physical fatigue during exercise.

**Methods:**

A randomized, double-blind, placebo-controlled, crossover study was carried out by 12 healthy adults. The participants were randomly assigned to receive acute oral administration of either 250 mL of the mangosteen-based juice (supplementation treatment; 305 mg of α-mangostin and 278 mg of hydroxycitric acid) or a placebo (control treatment) 1 h before cycle ergometer exercise. Time to exhaustion, heart rate, Borg Rating of Perceived Exertion score, blood biochemical markers (namely ammonia, cortisol, creatine kinase, aspartate aminotransferase, alanine aminotransferase, glucose, and lactate), muscle dynamic stiffness, and Profile of Mood States (POMS) were evaluated and recorded.

**Results:**

The results showed all parameters we examined were significantly altered by the exercise challenge, which demonstrated they directly reflected the condition of fatigue. However, there were no differences between the two treatments besides a positive impact on the POMS examination.

**Conclusions:**

The occurrence of physical fatigue depends on multiple underlying mechanisms. We concluded that acute mangosteen supplementation had no impact on alleviating physical fatigue during exercise.

## Background

Fatigue can be classified as either mental or physical. Physical fatigue, also called peripheral fatigue or muscle fatigue [1], is defined as a decrease of force-generating capacity. It develops gradually during exercise and occurs when the required force or exercise intensity can no longer be maintained [2]. Unsatisfactory exercise performance is mainly due to the occurrence of physical fatigue. Fatigue may also be associated with a shortened duration of exercise or diminishment in the degree of aerobic activity and can lead to an increased risk for injury [3]. There is no single mechanism of fatigue; however, the most important mechanisms that cause muscle fatigue include: (1) overproduction of reactive oxygen species (ROS) and (2) acidosis and depletion of adenosine triphosphate (ATP) due to increased consumption or decreased provision [4]. Therefore, identifying methods of eliminating oxidative stress and promoting balanced energy expenditure in order to reduce fatigue is a topic that has fascinated researchers in the sports nutrition field.

**Fig. 1:**
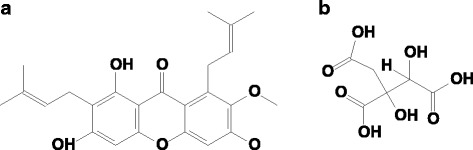
Chemical structure. **a** α-mangostin. **b** hydroxycitric acid

The purple mangosteen (*Garcinia mangostana*), known as the "queen of fruit," is widely consumed and unique not only because of its outstanding appearance and flavor but also its remarkable and diverse pharmacological effects, such as antioxidant, anti-inflammatory, and antimicrobial activities [5]. In Southeast Asia, mangosteen has been used as traditional medicine to treat skin infections, wounds, dysentery, and urinary tract infections [6, 7]. The main components of mangosteen are xanthones (e.g., α-mangostin (Fig. 1(a))). Mangosteen has been shown to scavenge 2,2-diphenyl-1-picrylhydrazyl (DPPH) radicals [8], diminish thiobarbituric reactive substances (TBARS) [9], and reduce ROS production (e.g. H_2_O_2_, O^2−^ and ONOO^−^) [10–12]. Devi et al. [13] revealed the protective effects of α-mangostin on lipid peroxidation and the antioxidant tissue defense system through reduction of the activities of superoxide dismutase, catalase, and glutathione peroxidase in rats with myocardial infarction. Another active compound existing in the *Garcinia* genus is hydroxycitric acid (HCA) (Fig. 1(b)). HCA is a competitive inhibitor of ATP citrate lyase and may reduce the synthesis of cytosolic acetyl-coenzyme A, which plays a key role in lipogenesis and cholesterogenesis [14]. Short- and long-term intake of HCA increases fat oxidation [15–17] and promotes the utilization of fatty acid as an energy source by decreasing the respiratory exchange ratio (RER) [18]. Additionally, previous studies have revealed that HCA intake enhanced glycogen synthesis in exercised human skeletal muscle [19], but reduced glycogen utilization [20]. Although the literature suggests a potential effect of mangosteen for alleviating sports-induced physical fatigue, there is no scientific data directly testing its benefits.

In the present study, we used a randomized, double-blind, placebo-controlled, crossover design to test the effects of acute mangosteen supplementation on exercise-induced physical fatigue in healthy subjects. In addition to measuring muscle elasticity characteristics with the MyotonPRO device, we focused on examining biomarkers that directly reflect the condition of physical fatigue that occurs during exercise, including ammonia, aspartate aminotransferase (AST), alanine aminotransferase (ALT), creatine kinase (CK), and lactate [21]. We also applied Borg Rating of Perceived Exertion (RPE) and Profile of Mood States (POMS) to measure physical activity intensity and the condition of vigor as well as fatigue.

## Methods

### Liquid chromatographic analysis of mangosteen-based juice

A commercially available mangosteen-based functional juice (Lord Duke Biotechnology Corporation, Taipei, Taiwan) was employed as a supplemental treatment in the present study. The mangosteen-based juice is a liquid beverage containing 50 % whole-fruit mangosteen, 7 % pomegranate juice concentrate, 7 % acerola juice concentrate, 12 % white grape juice concentrate, 6 % strawberry juice concentrate, 10 % passion fruit juice concentrate and 8 % tamarind jams blend. High-performance liquid chromatography (HPLC) was used to determine the major components of the mangosteen-based juice. HPLC-UV analysis was conducted using an HPLC system (Hitachi, Tokyo, Japan) consisting of a L-2420 UV–Vis detector, a L-2200 autosampler, and a L-2130 pump. LiChroCART^®^ 250–4,6 (Merck KGaA, Darmstadt, Germany) was used as an analytical column. The injection volume was 20 μL. For determination of the content of α-mangostin, the mobile phase, composed of solvent A (methanol) and solvent B (0.15 % formic acid) in a ratio of 2:98, was delivered at a flow rate of 1 mL · min^−1^. The detection UV wavelengths were set at 254 nm. For determination of the HCA content, the mobile phase, composed of solvent A (methanol) and solvent B (0.1 % H_3_PO_4_) in a ratio of 98:2, was delivered at a flow rate of 1 mL · min^−1^. The detection UV wavelengths were set at 220 nm. All of the analyses were performed in triplicate.

### Participants

In total, 12 healthy, physically active adults (6 male and 6 female, age = 22.6 ± 1.1 y, height = 171.3 ± 10.5 cm, weight = 63.6 ± 11.2 kg, body mass index = 21.7 ± 3.2 kg · m^−2^) volunteered to participate in this study. Each volunteer completed a health questionnaire to exclude participants at risk for or with preexisting cardiovascular diseases, diabetes, or other high-risk medical conditions. Volunteers also could not be taking any regular supplements or medications. The study procedures were reviewed and approved by the Institutional Review Board of Kaohsiung Medical University Chung-Ho Memorial Hospital (No. KMUHIRB-2014-11-09). All participants provided written informed consent after receiving an explanation of the experimental procedures.

### Experimental design

**Fig. 2:**
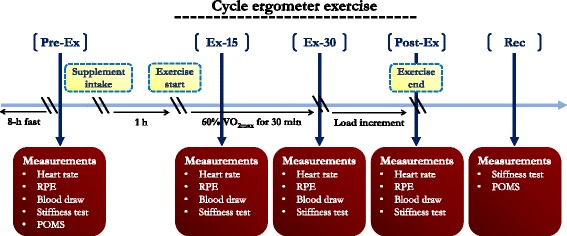
Illustration of the experimental design

The participants were randomly assigned in a double-blind crossover design to receive a single dose of either 250 mL of the mangosteen-based juice (supplementation treatment) or a placebo (the mangosteen-based juice replaced 50 % whole-fruit mangosteen with pure water; control treatment) in a random order. In order to have a placebo juice that was indistinguishable for participants, it was necessary to supply a juice blend that was similar in flavor and color. Therefore, the only difference between the supplementation and the placebo treatment is the removal of the 50 % whole-fruit mangosteen from the placebo. The order was counterbalanced between 2 treatments. The washout period was 2 weeks. After an 8-h fast, the supplement was administered 1 h before cycle ergometer exercise (Fig. 2). During the experiments, the participants wore a heart rate monitor attached to an elastic strap (Polar Electro, Kempele, Finland) around the chest. A small catheter was inserted into an antecubital vein for venous blood sampling. The heart rate and RPE score were recorded and blood was collected before exercise (Pre-Ex), at 15 min (15-Ex) and 30 min (30-Ex) time points during exercise, and immediately after exercise (Post-Ex). Muscle dynamic stiffness was measured at Pre-Ex, 15-Ex, 30-Ex, and 15 min after exercise (Rec). The POMS was administered at Pre-Ex and Rec.

**Fig. 3:**
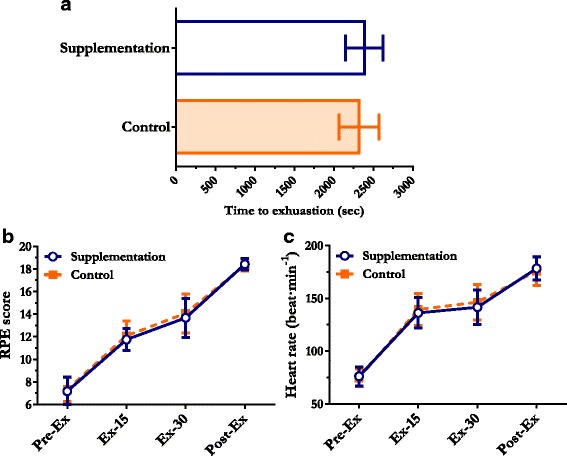
Effect of mangosteen supplementation on time to exhaustion, RPE, and heart rate. **a** time to exhaustion. **b** RPE. **c** heart rate. Data are expressed as the mean (±standard deviation). Pre-Ex; before exercise, 15-Ex; 15 min during exercise, 30-Ex; 30 min during exercise, Post-Ex; immediately after exercise

### Cycle ergometer exercise protocol

In a preliminary test, the predicted maximal oxygen consumption (VO_2max_) of each participant was calculated. The test was performed on a Monark Ergomedic 839 E cycle ergometer (Monark Exercise AB, Vansbro, Sweden) equipped with adjustable seat, handlebars, and pedals. After a 5-min warm-up, the Åstrand protocol [22] was performed. For male participants, the initial workload started from 100 W and increased by 50 W every 2 min; for female participants, the load started from 50 W and increased by 25 W every 2 min. The participants were asked to maintain the frequency of 50 rpm. The end of the test was defined as the attainment of at least 2 of the 3 following criteria: (1) no longer maintaining the required pedaling frequency, (2) heart rate within 10 beats of the age-predicted maximum, and (3) RPE greater than 18.

The predicted VO_2max_ of each participant was calculated using the following equation [22]:

$$ \mathrm{V}{\mathrm{O}}_2=\mathrm{W}/\mathrm{M}\times 1.8+3.5+3.5 $$

W = work rate in kilograms per minute, M = body mass in kilograms.

In the main exercise test, each participant performed with a relative work rate of 60 % of VO_2max_ on the same cycle ergometer for 30 min. At 30 min, the workload increased incrementally by 15 W every 2 min until exhaustion.

### Analysis of blood biochemical markers

At each blood collection, blood glucose and lactate were measured using a ONETOUCH^®^ UltraEasy™ blood glucose meter (LifeScan, Milpitas, CA, USA) and Lactate Pro™ blood lactate test meter (ARKRAY, Kyoto, Japan), respectively. Remaining blood was splitted between tubes containing a clot activator and K_2_EDTA and centrifuged at 2000 × *g* and 4 °C for 10 min to obtain serum and plasma samples, respectively. Plasma ammonia, serum CK, AST, and ALT were determined using a Toshiba TBA™-c16000 Chemistry Analyzer (Toshiba Medical Systems Corporation, Tochigi-ken, Japan). Serum cortisol was determined using an ARCHITECT i2000SR Immunoassay Analyzer (Abbott Laboratories. Abbott Park, Illinois, USA).

### Measurement of muscle dynamic stiffness

Muscle elasticity characteristic was measured using a handheld MyotonPRO device (Myoton AS, Tallinn, Estonia). The quadriceps femoris of the participants was tested in the middle point of the muscle belly on the right body side while the knee remained perpendicular to the pedal. The dynamic stiffness of the muscle was calculated and represented the ability of tissue to restore its shape after removal of an external force acting on the muscle.

### Profile of Mood States

The POMS is a 65-item questionnaire [23] that was used to evaluate the psychological conditions of the participants. The overall mood, including tension, depression, anger, vigor, and confusion, was examined. The participants self-evaluated their feelings at Pre-Ex and Rec. Finally, the scores of the POMS were calculated.

### Statistical analysis

SPSS 20.0 (International Business Machines Corporation, Armonk, NY, USA) was used for statistical analyses. Data are expressed as the mean (±standard deviation). The effects of treatments, times, and their interaction on blood biochemical markers as well as muscle dynamic stiffness and the POMS were analyzed with repeated measures through analysis of variance (ANOVA) and corrected with Bonferroni's *post*-*hoc* multiple comparisons. The Student's paired *t*-test was used to further compare all characteristics between treatments. A *P* value < 0.05 was considered statistically significant.

## Results

### Analysis of the mangosteen-based juice

The proposed HPLC method maintained good linearity in the calibration curve from 2 to 50 (*R*^2^ = 0.9998) and 5 to 100 μg · mL^−1^ (*R*^2^ = 0.9999) for α-mangostin and HCA, respectively. The concentration of α-mangostin and HCA of the mangosteen juice was 1.22 (0.02) and 1.11 (0.01) mg · mL^−1^, respectively.

### Effect of mangosteen supplementation on time to exhaustion, RPE, and heart rate

Mangosteen supplementation increased the time to exhaustion by 13.3 % in the incremental workload period compared to control treatment; however, there were no significant differences in total time to exhaustion between the treatments (Fig. 3a) (*P* = 0.128).

RPE score was progressively elevated in both treatments, indicating the exercise protocol was applied properly. However, the RPE score of each treatment did not differ among the time points (Fig. 3b) (Pre-Ex: *P* = 0.689, Ex-15: *P* = 0.305, Ex-30: *P* = 0.508, Post-Ex: *P* = 0.586).

The heart rate of each treatment showed no differences at any time point (Fig. 3c) (Pre-Ex: *P* = 0.415, Ex-15: *P* = 0.109, Ex-30: *P* = 0.059, Post-Ex: *P* = 0.327).

### Effect of mangosteen supplementation on blood biochemical markers

A significant time effect (*P* < 0.05) and a lack of a treatment effect as well as a treatment by time interaction (*P* > 0.05) were documented for blood biochemical markers (Table 1). At Ex-15, the levels of ammonia, CK, and lactate were markedly elevated (*P* < 0.05 vs. Pre-Ex), whereas the levels of cortisol and glucose were decreased (*P* < 0.05 vs. Pre-Ex). At Ex-30, the levels of ammonia, CK, ALT, and lactate were markedly elevated (*P* < 0.05 vs. Pre-Ex), whereas the levels of cortisol and glucose were decreased (*P* < 0.05 vs. Pre-Ex). At Post-Ex, the levels of ammonia, CK, AST, ALT, and lactate were markedly elevated (*P* < 0.05 vs. Pre-Ex), whereas the levels of cortisol were decreased (*P* < 0.05 vs. Pre-Ex).

**Table 1:** Effect of mangosteen supplementation on blood biochemical markers

Marker	Treatment	Time				Treatment	Time	Treatment × Time
		Pre-Ex	Ex-15	Ex-30	Post-Ex			
Ammonia (μmol · L^−1^)	Control	85.3 (20.8)	111.7** (34.2)	120.1** (45.6)	165.4** (51.8)	0.629	0.000	0.918
	Supplementation	94.5 (36.5)	115.3** (39.3)	127.8** (46.2)	167.9** (65.6)			
Cortisol (μg · dL^−1^)	Control	10.91 (3.32)	7.33** (2.21)	7.23** (2.29)	7.57** (2.46)	0.348	0.000	0.486
	Supplementation	12.04 (2.71)	7.45** (1.98)	7.12** (2.33)	7.77** (2.72)			
CK (U · L^−1^)	Control	109.2 (53.6)	118.5** (56.1)	121.3** (56.2)	128.9** (61.3)	0.821	0.001	0.273
	Supplementation	116.6 (61.5)	124.2** (67.1)	125.3** (66.7)	127.6** (60.2)			
AST (U · L^−1^)	Control	23.9 (15.7)	26.3 (15.9)	26.7 (16.6)	29.6** (18.3)	0.279	0.000	0.795
	Supplementation	18.7 (4.8)	21.1 (5.3)	20.8 (4.9)	24.5** (8.4)			
ALT (U · L^−1^)	Control	17.3 (12.2)	18.3 (13.0)	18.3** (13.0)	19.4** (14.5)	0.095	0.006	0.756
	Supplementation	13.1 (7.5)	13.8 (7.9)	14.2** (7.7)	14.9** (9.2)			
Glucose (mg · dL^−1^)	Control	88.0 (7.4)	73.4** (11.6)	72.3** (9.3)	81.7 (13.9)	0.250	0.000	0.172
	Supplementation	86.3 (7.8)	69.3*^,^** (10.5)	70.8** (7.8)	83.5 (10.5)			
Lactate (mmol · L^−1^)	Control	1.03 (0.25)	3.22** (1.22)	3.20** (1.39)	7.61** (1.65)	0.775	0.000	0.260
	Supplementation	1.02 (0.20)	3.01** (1.16)	2.93** (1.22)	8.21** (1.60)			

*Note*: Data are expressed as the mean (±standard deviation). *P < 0.05 indicates significant differences between the treatments (analyzed using the Student's paired *t*-test). **P < 0.05 indicated significant differences compared to Pre-Ex (analyzed using repeated measures through ANOVA and corrected with Bonferroni's *post*-*hoc* multiple comparisons)

*Pre*-*Ex* before exercise, *15*-*Ex* 15 min during exercise, *30*-*Ex* 30 min during exercise, *Post*-*Ex* immediately after exercise, *CK* creatine kinase, *AST* aspartate transaminase, *ALT* alanine transaminase

When we further examined the effects of the mangosteen supplemented intervention according to the Student's paired *t*-test, there were no significant differences in the levels of blood biochemical markers among Pre-Ex (ammonia: *P* = 0.448, cortisol: *P* = 0.285, CK: *P* = 0.674, AST: *P* = 0.269, ALT: *P* = 0.085, glucose: *P* = 0.431, lactate: *P* = 0.818), Ex-15 (ammonia: *P* = 0.759, cortisol: *P* = 0.736, CK: *P* = 0.760, AST: *P* = 0.263, ALT: *P* = 0.084, lactate: *P* = 0.562), Ex-30 (ammonia: *P* = 0.548, cortisol: *P* = 0.803, CK: *P* = 0.822, AST: *P* = 0.233, ALT: *P* = 0.103, glucose: *P* = 0.456, lactate: *P* = 0.393), and Post-Ex (ammonia: *P* = 0.887, cortisol: *P* = 0.786, CK: *P* = 0.936, AST: *P* = 0.366, ALT: *P* = 0.115, glucose: *P* = 0.372, lactate: *P* = 0.252) between the 2 treatments. However, mangosteen supplementation caused a slight but noteworthy decrease of blood glucose at Ex-15 (*P* = 0.036).

### Effect of mangosteen supplementation on muscle dynamic stiffness

The muscle dynamic stiffness significantly changed among the time points (*P* < 0.05) (Table 2). It remarkably increased by 18.6─27.6 % during exercise and reversed to baseline at Rec. However, no differences were evident in the treatments and treatment by time interaction (*P* > 0.05). Furthermore, the results of the Student's paired *t*-test showed no significant differences between the two treatments among the time points (Pre-Ex: *P* = 0.467, Ex-15: *P* = 0.902, Ex-30: *P* = 0.312, Post-Ex: *P* = 0.765, Rec: *P* = 0.951).

**Table 2:** Effect of mangosteen supplementation on muscle dynamic stiffness

Marker	Treatment	Time					Treatment	Time	Treatment × Time
		Pre-Ex	Ex-15	Ex-30	Post-Ex	Rec			
Dynamic stiffness (N · m^−1^)	Control	308.0 (50.6)	378.0** (68.4)	365.2** (54.9)	392.9** (65.7)	315.4 (59.3)	0.860	0.000	0.512
	Supplementation	315.2 (45.2)	375.5** (46.9)	381.8** (43.8)	387.1** (54.2)	314.0 (59.3)			

*Note*: Data are expressed as the mean (±standard deviation). **P < 0.05 indicated significant differences compared to Pre-Ex (analyzed using repeated measures through ANOVA and corrected with Bonferroni's *post*-*hoc* multiple comparisons)

*Pre*-*Ex* before exercise, *15*-*Ex* 15 min during exercise, *30*-*Ex* 30 min during exercise, *Post*-*Ex* immediately after exercise, *Rec* 15 min after exercise

### Effect of mangosteen supplementation on Profile of Mood States

None of the parameters of POMS differed between the treatments (*P* > 0.05) (Table 3). The score of vigor significantly decreased and the score of fatigue significantly increased after exercise (*P* < 0.05), while others remained unchanged (*P* > 0.05). The scores of fatigue were significantly different in the treatment by time interaction (*P* < 0.05), while others remained unchanged (*P* > 0.05). According to the Student's paired *t*-test, the effect of supplementation and the placebo on the POMS did not significantly differ at Pre-Ex (tension: *P* = 0.196, depression: *P* = 0.627, anger: *P* = 0.586, vigor: *P* = 0.698, fatigue: *P* = 0.921, confusion: *P* = 0.139) or Rec (tension: *P* = 0.082, depression: *P* = 0.236, anger: *P* = 0.104, vigor: *P* = 0.300, confusion: *P* = 0.384); however, we observed a significantly lower score for fatigue in the supplementation treatment (*P* = 0.023).

**Table 3:** Effect of mangosteen supplementation on Profile of Mood States

Marker	Treatment	Time		Treatment	Time	Treatment × Time
		Pre-Ex	Rec			
Tension	Control	2.8 (2.2)	2.5 (1.7)	0.126	0.473	0.884
	Supplementation	2.2 (1.7)	1.8 (1.8)			
Depression	Control	2.4 (1.7)	2.3 (1.4)	0.404	0.497	0.761
	Supplementation	2.1 (1.9)	1.8 (1.4)			
Anger	Control	0.4 (0.9)	0.3 (0.9)	0.389	0.723	0.054
	Supplementation	0.3 (0.9)	0.6 (1.4)			
Vigor	Control	11.4 (3.5)	7.7** (4.0)	0.319	0.001	0.559
	Supplementation	11.8 (3.8)	8.8** (4.2)			
Fatigue	Control	2.7 (3.1)	13.7** (4.3)	0.124	0.000	0.001
	Supplementation	2.6 (2.9)	10.6*^,^** (4.6)			
Confusion	Control	2.8 (1.8)	3.9 (3.5)	0.818	0.333	0.087
	Supplementation	3.3 (1.7)	3.2 (2.1)			

*Note*: Data are expressed as the mean (±standard deviation). *P < 0.05 indicates significant differences between the treatments (analyzed using the Student's paired *t*-test). **P < 0.05 indicated significant differences compared to Pre-Ex (analyzed using repeated measures through ANOVA and corrected with Bonferroni's *post*-*hoc* multiple comparisons)

*Pre*-*Ex* before exercise, *Rec* 15 min after exercise

## Discussion

The *C*_max_ of α-mangostin in plasma appeared 60 min after administration [24]. Kondo et al. [24] administered acute 59 mL mangosteen product (containing 94.2 mg of mangostins) and found an increase in oxygen radical absorbance capacity (ORAC). Udani et al. [25] administered 6─18 oz mangosteen juice twice a day for 8 weeks and showed a decrease in C-reactive protein. Xie et al. [26] administered a single dose of 245 mL of xanthone-rich beverage and also found an increase in ORAC. Likewise, the *C*_max_ of HCA in plasma appeared 60–120 min after administration [14]. Van Loon et al. [14] administered 18000 mg of HCA before exercise and observed a decrease in plasma lactate concentrations at the 30 min time point during exercise. Lim et al. [20] administered 250 mg of HCA for 5 d prior to exercise and observed that the treatment enhanced exercise time to exhaustion and reduced the RER and carbohydrate oxidation. In our study, we chose to administer an acute dose of 250 mL mangosteen-based juice (approximately containing 305 mg of α-mangostin and 278 mg of HCA) 1 h before the exercise challenge.

Heart rate monitoring is also used to determine the exercise intensity [27] and predict perceived exertion and mood [28]. A low resting heart rate, diminished increase in heart rate during exercise, and diminished decrease in heart rate during recovery indicate a high risk for myocardial infarction [29]. In our study, we did not find the differences of the time to exhaustion, RPE, and heart rate existed between mangosteen supplementation and control treatment.

Biochemical variables, including ammonia, CK, AST, ALT, and lactate, are important indicators of muscle fatigue after exercise [30–32]. Our results revealed that blood ammonia, CK, AST, ALT, and lactate progressively increased during both exercise sessions. Particularly, the most sensitive markers altered by exercise were ammonia and lactate. Ammonium ions are a toxic waste product of metabolism. If the consumption of ATP exceeds the supply, the body transfers adenosine diphosphate (ADP) to ATP and adenosine monophosphate (AMP). AMP is subsequently degraded to inosine monophosphate and ammonia [33]. Ammonia closely follows the lactate response during exercise [34]. When the oxidative phosphorylation of ADP to generate ATP fails to meet the requirement, which is the case under acidic conditions, the production of ATP shifts from aerobic to anaerobic processes [34]. Elevated serum lactate reflects that the aerobic ATP generation is insufficient for requirement and needs to be supplemented with anaerobic ATP generation. The lactate threshold is the percentage of the maximal exercise intensity at which the concentration of lactate begins to exponentially increase [35]. The results showed that the exercise protocol employed led the participants to reach lactate threshold.

On the other hand, cortisol is released from the adrenal cortex in response to physical and mental stress [36] and is thought to allow the athletes to restrain the stress response under physical loading [37]. Interestingly, our study found that cortisol dropped after exercise began. In humans, cortisol undergoes diurnal variation; the peak level presents in the early morning and the lowest level presents around midnight. Because our experiment began in the morning when the participants were fasted, it would be expected that cortisol would reach peak levels at Pre-Ex. In addition, we observed caused a slight but noteworthy decrease of blood glucose at Ex-15 in mangosteen supplementation treatment. Increasing blood glucose and insulin concentrations during exercise has been found to spare muscle glycogen and increase aerobic endurance [38]. It remains doubtful that the decrease in glucose level entails a safety concern.

The elasticity characteristics of the quadriceps femoris were measured using a handheld MyotonPRO device, which is a feasible and noninvasive approach. Muscle elasticity is defined as the ability of a muscle to return to its original form or shape after the removal of a deforming force, and muscle stiffness is a muscle’s resistance to deformation [39]. Decreased elasticity results in dynamic stiffness and quicker muscle fatigue. Our results showed the muscle dynamic stiffness significantly during exercise period; however, it did not differ between supplementation and control treatment. Regarding the POMS, a common instrument for measuring fatigue, has been shown to be more responsive to changes in fatigue than other questionnaires [40]. We observed a significant amelioration of fatigue score in the POMS of the supplementation treatment.

## Conclusions

The occurrence of physical fatigue depends on multiple underlying mechanisms. Supplementation of mangosteen had no significant effect on reducing the physical fatigue that occurs during exercise. Although the single dose supplementation did not reduce physical fatigue, it did have a positive impact on POMS scores. Based on this and previous evidences that chronic supplementation of mangosteen has a beneficial effect on substrate metabolism, future studies should explore the impact of long-term supplementation on exercise-induced fatigue.
